# A Putative Role of Teneurin-2 and Its Related Proteins in Astrocytes

**DOI:** 10.3389/fnins.2019.00655

**Published:** 2019-06-27

**Authors:** Gestter W. L. Tessarin, Ola M. Michalec, Kelly R. Torres-da-Silva, André V. Da Silva, Roelf J. Cruz-Rizzolo, Alaide Gonçalves, Daniele C. Gasparini, José A. C. Horta-Júnior, Edilson Ervolino, Jackson C. Bittencourt, David A. Lovejoy, Cláudio A. Casatti

**Affiliations:** ^1^Department of Basic Sciences, School of Dentistry of Araçatuba, São Paulo State University (UNESP), Araçatuba, Brazil; ^2^Department of Anatomy, Institute of Biosciences of Botucatu, São Paulo State University (UNESP), Botucatu, Brazil; ^3^Department of Cell and Systems Biology, University of Toronto, Toronto, ON, Canada; ^4^School of Medicine, Federal University of Mato Grosso do Sul (UFMS), Três Lagoas, Brazil; ^5^Department of Anatomy, Institute of Biomedical Sciences, São Paulo University (USP), São Paulo, Brazil

**Keywords:** teneurin, teneurin c-terminal associated peptide, latrophilin, mechanical brain injury, cerebral cortex, reactive astrocytes, adult rat

## Abstract

Teneurins are type II transmembrane proteins comprised of four phylogenetically conserved homologs (Ten-1-4) that are highly expressed during neurogenesis. An additional bioactive peptide named teneurin C-terminal-associated peptide (TCAP-1-4) is present at the carboxyl terminal of teneurins. The possible correlation between the Ten/TCAP system and brain injuries has not been explored yet. Thus, this study examined the expression of these proteins in the cerebral cortex after mechanical brain injury. Adult rats were subjected to cerebral cortex injury by needle-insertion lesion and sacrificed at various time points. This was followed by analysis of the lesion area by immunohistochemistry and conventional RT-PCR techniques. Control animals (no brain injury) showed only discrete Ten-2-like immunoreactive pyramidal neurons in the cerebral cortex. In contrast, Ten-2 immunoreactivity was significantly up-regulated in the reactive astrocytes in all brain-injured groups (*p* < 0.0001) when compared to the control group. Interestingly, reactive astrocytes also showed intense immunoreactivity to LPHN-1, an endogenous receptor for the Ten-2 splice variant named Lasso. Semi-quantitative analysis of Ten-2 and TCAP-2 expression revealed significant increases of both at 48 h, 3 days and 5 days (*p* < 0.0001) after brain injury compared to the remaining groups. Immortalized cerebellar astrocytes were also evaluated for Ten/TCAP expression and intracellular calcium signaling by fluorescence microscopy after TCAP-1 treatment. Immortalized astrocytes expressed additional Ten/TCAP homologs and exhibited significant increases in intracellular calcium concentrations after TCAP-1 treatment. This study is the first to demonstrate that Ten-2/TCAP-2 and LPHN-1 are upregulated in reactive astrocytes after a mechanical brain injury. Immortalized cerebellar astrocytes expressed Ten/TCAP homologs and TCAP-1 treatment stimulated intracellular calcium signaling. These findings disclose a new functional role of the Ten/TCAP system in astrocytes during tissue repair of the CNS.

## Introduction

Teneurins are type II transmembrane glycoproteins composed of four paralogues (Ten-1-4), mainly expressed during central nervous system (CNS) development (Baumgartner and Chiquet-Ehrismann, 1993; Baumgartner et al., 1994; Levine et al., 1994; Rubin et al., 1999; Tucker and Chiquet-Ehrismann, 2006). Teneurins encompass around 2800 amino acids, with the N-terminal intracellular domain consisting of approximately 300–375 amino acids, which can be cleaved and translocated to the nucleus acting as a transcription factor or can mediate cytoskeletal interactions (Bagutti et al., 2003; Nunes et al., 2005; Tucker and Chiquet-Ehrismann, 2006). The transmembrane and C-terminal extracellular domains comprise 34 and 2400 amino acids, respectively (Bagutti et al., 2003; Nunes et al., 2005; Tucker and Chiquet-Ehrismann, 2006). The extracellular domain contains several sites for homophilic or heterophilic interactions and additional potential cleavage sites that can generate soluble signaling molecules (Bagutti et al., 2003; Nunes et al., 2005; Lovejoy et al., 2006; Tucker and Chiquet-Ehrismann, 2006; Tucker et al., 2012; Mosca, 2015). Splice variants of the teneurins are also found in vertebrates, transcribing some proteins such as a Ten-2 related protein named Lasso (latrophilin1-associated synaptic surface organizer), an endogenous ligand for the G-protein-coupled receptor named latrophilin (LPHN) (Silva et al., 2011; Tucker et al., 2012; Boucard et al., 2014). Latrophilins are constituted by three isoforms (LPHN-1-3), also known as Adhesion G Protein-Coupled Receptor, subfamily L (ADGRL1-3) (Meza-Aguilar and Boucard, 2014). Lasso can also undergo intracellular cleavage resulting in soluble molecules, which are secreted and may interact with LPHN in other cells (Vysokov et al., 2016). Recently, a study demonstrated similarities of the Ten-2 carboxyl terminal to bacterial Tc-toxins (Li et al., 2018). This region shows complex arrangements, permitting an heterophilic interaction with LPHN, which controls intracellular cyclic AMP (cAMP) (Li et al., 2018).

Teneurins are primarily involved with neuronal migration, axonal guidance, as well as formation, differentiation and maintenance of synapses in the CNS (Mosca, 2015; Antinucci et al., 2016). Previous studies have also correlated these proteins with mental disorders, congenital diseases and some types of tumors (Vinatzer et al., 2008; Ziegler et al., 2012; Boeva et al., 2013; Heinrich et al., 2013; Nakaya et al., 2013; Ivorra et al., 2014; Zhang et al., 2014; Bastías-Candia et al., 2015; Hor et al., 2015; Lovejoy and Pavlović, 2015; Schöler et al., 2015; Vater et al., 2015; Alkelai et al., 2016; Cheng et al., 2017; Graumann et al., 2017; Talamillo et al., 2017). Interestingly, teneurins contain a cleavage site in their carboxyl terminal that originates fragments of 40 to 41 amino acids, named teneurin C-terminal-associated peptides (TCAP-1-4), which show structural similarities to corticotrophin-releasing factor (CRF) (Qian et al., 2004; Wang et al., 2005; Lovejoy et al., 2006). *In vitro* studies using immortalized neurons have demonstrated that TCAP-1 stimulates neurite outgrowth, regulates brain-derived neurotrophic factor (BDNF) and acts as a neuroprotective agent (Al Chawaf et al., 2007a; Trubiani et al., 2007; Ng et al., 2012). *In vivo* studies in rats established that TCAP-1 treatment modulates dendritic morphology in hippocampal neurons and reduces FOS induction in neurons in limbic regions, stimulated by CRF intracerebral administration (Al Chawaf et al., 2007b; Tan et al., 2009, 2011). TCAP-1 also reduces stress-related behaviors and eliminates cocaine-seeking reinstatement in adult rats (Wang et al., 2005; Al Chawaf et al., 2007b; Kupferschmidt et al., 2011; Tan et al., 2011; Erb et al., 2014). A novel finding reported significantly increased glucose uptake in the rat brain 3 days after a single subcutaneous TCAP-1 injection, as well as decreased blood glucose 1 week later (Hogg et al., 2018). *In vitro* data corroborated the TCAP-1 action in the glucose metabolism in neurons, indicating that TCAP-1 can represent a peptide signaling substance that regulates glucose uptake, regardless of insulin-mediated glucose regulation (Hogg et al., 2018).

Preliminary unpublished screening assays performed in our laboratory indicated that Ten-2/TCAP-2 showed substantial changes in experimental brain disorders induced in adult rats (Tessarin and Casatti, unpublished data). Thus, we focused mainly on the correlations between Ten-2/TCAP-2 and reactive astrocytes. For this, controlled mechanical brain injury was induced by a metal needle insertion lesion in the cerebral cortex of adult rats, followed by immunohistochemistry and conventional RT-PCR analysis. Additionally, LPHN immunoreactivity was qualitatively analyzed by immunohistochemistry. In order to adopt an *in vitro* model for further studies, immortalized mouse cerebellar astrocytes were also characterized for Ten/TCAP homolog expressions and evaluated concerning calcium-signaling modulation after TCAP-1 treatment.

## Materials and Methods

### *In vivo* Study

#### Animals

Forty-five adult male Wistar rats (280–300 g) were obtained from the central animal facility at the School of Dentistry of Araçatuba (UNESP, Araçatuba, SP, Brazil) and maintained in the experimental room of the Morphology division of the Department of Basic Sciences for 15 days for environmental adaptation. The rats were kept in a 12/12 dark-light cycle (lights on at 7:00 am) under controlled temperature (23 ± 1°C) and humidity (50–65%), as well as water and rat chow *ad libitum*. The experimental protocols for animal handling and care were approved by the Institutional Committee of Animal Welfare (CEUA, process number 2015-00318). All efforts were made to reduce the number of animals and to minimize suffering.

#### Surgery Procedures

Animals were anesthetized by intramuscular injection of ketamine (80 mg/ kg; Virbac, SP, Brazil) and xylazine (10 mg/ kg; Bayer, RS, Brazil), and then positioned in stereotaxic apparatus, where the scalp was incised along the midline using a scalpel blade. The brain was exposed after drilling, using a spherical bur coupled to a high-speed rotation handpiece. The dura-mater was discretely incisioned to expose the cerebral cortex. For mechanical brain injury, a needle-insertion injury was created through vertical insertion of a sterile metal needle (0.8 mm diameter) maintained for 5 s in the cerebral cortex. The coordinates for the cerebral cortex lesion were 6.63 mm (rostro-caudal axis), 1.5 mm (mediolateral) and 4 mm (dorsoventral, from the cortical surface) (Paxinos and Watson, 1998). After surgery, animals were kept in individual cages and divided into groups of 24 h, 48 h, 3 and 5 days of postoperative periods (*n* = 9 per experimental time, considering *n* = 5 for immunohistochemistry and *n* = 4 for conventional RT-PCR analysis). Rats from the control group (*n* = 9, considering *n* = 5 for immunohistochemistry and *n* = 4 for conventional RT-PCR analysis) were subjected to brain exposure with no dura-mater or brain injury (sham-surgery: control group).

### Immunohistochemistry

#### Tissue Preparation

Animals were anesthetized as previously described and transcardially perfused with heparinized saline solution at room temperature (RT) (100–150 mL) followed by cold fixative solution (1,000 mL) containing 4% formaldehyde (obtained from paraformaldehyde heated to 65°C, #P6148, Sigma-Aldrich, MO, USA) diluted in phosphate buffer saline 0.1 M (PBS; pH 7.4). The brains were dissected and post-fixed in the same fixative solution for 4 h at 4°C. Subsequently, the brains were cryoprotected in PBS with 30% sucrose (#1894-1, Dinâmica, SP, Brazil) overnight at 4°C. Coronal 30 μm-thick histological sections were obtained in a freezing microtome (SM 2000R, Leica, HE, Germany) and stored in 12-well culture plates with anti-freezing solution (PB 0.025 M, NaCl 0.225%, sucrose 15% and ethylene glycol 35%) at −20°C for further processing.

### Immunoperoxidase Staining

Histological sections were submitted to immunoperoxidase staining method to obtain a detailed morphology of the neural cells exhibiting immunoreactivity to Ten-2. Initially, one series of histological sections (360 μm intersection interval) was washed (3 × 10 min) in PBS and submitted to peroxidase endogenous inhibition using 0.3% hydrogen peroxide (#H3410, Sigma-Aldrich, MO, USA) in PBS, for 30 min at RT. Next, the sections were washed (3 × 10 min) in PBS and blocked for non-specific bindings using 5% non-fat milk in PBS with 0.03% triton X-100 (PBS-T, #100882547, X-100, Sigma-Aldrich, MO, USA), followed by 3% bovine serum albumin (A9647, Sigma-Aldrich, MO, USA) in PBS-T, for 1 h at RT for each blocking. Additional blocking was performed using 2% normal donkey serum (#017-000-121, Jackson Immunoresearch, PA, USA) in PBS-T, overnight at 4°C. Sections were initially incubated in primary polyclonal antibody anti-Ten-1-4 (Ten-1, 1:250, H00010178-A01, lot # 07310, ABNOVA, Taipei, Taiwan; Ten-2, 1:100, Lot # K1910, sc-165674, Santa Cruz Biotechnology, CA, USA; Ten-3, 1:500, Lot # B0910, sc 136918, Santa Cruz Biotechnology; Ten-4, 1:1000, Lot # B2610, sc-134883, Santa Cruz Biotechnology), diluted in PBS-T and 2% normal donkey serum, for 48 h at 4°C. Then, the sections were incubated in biotinylated secondary antibody (for Ten-1, 1;800, Lot # X0623, BA-9200, Vector Laboratories Inc., CA, USA; for Ten-2, 1:800, lot # G0815, sc-2042, Santa Cruz Biotechnology; for Ten-3 and Ten-4, 1:800, Lot # E2213, sc-2089, Santa Cruz Biotechnology), followed by avidin-biotin complex (1:500, PK-6100, Vector Laboratories Inc., CA, USA) in PBS-T, for 1 h at RT each step. The immunoreaction was visualized by developing the sections in 0.05% diaminobenzidine as a chromogen (DAB, #32741, Sigma-Aldrich, MO, USA) with nickel ammonium sulfate (#N48-500, Fisher Chemical, NJ, USA) and 0.3% hydrogen peroxidase, under light microscope analysis for reaction control. After that, the sections were mounted on gelatin-coated slides and maintained for approximately 24 h at 55–56°C in an oven. Finally, they were dehydrated in alcohol, cleared in xylenes and cover-slipped with DPX as a mounting medium (#06522, Sigma-Aldrich, MO, USA).

### Double Immunofluorescence

This method was performed for detection and counting of nuclear astrocyte profiles (DAPI; glial fibrillary acidic protein—GFAP) exhibiting immunoreactivity to Ten-2 or to qualitatively analyse latrophilin immunoreactivity in the experimental groups. For this, one series of histological sections was washed (3 × 10 min) in PBS and submitted to the same blocking steps for elimination of possible unspecific antibody interaction, as previously mentioned. Sections were incubated in primary polyclonal antibody anti-Ten-2 or LPHN1-3 (Ten-2, 1:100, Lot # K1910, sc-165674, Santa Cruz Biotechnology, CA, USA; LPHN-1, 1:200, D-20, Lot # I2909, sc-34484; LPHN-2, 1:200, A-14, Lot # H0608, sc-47091; LPHN-3, 1:200, P-17, Lot # A0907, sc-47095, Santa Cruz Biotechnology, CA, USA) diluted in PBS-T and 2% normal donkey serum, for 48 h at 4°C. Subsequently, sections were incubated in species-specific biotinylated secondary antibody, followed by Cy^3^-streptavidin (1:500, #016-160-084, Jackson Immunoresearch, PA, USA). The sections were then washed (3 × 10 min) in PBS and incubated in primary polyclonal antibody anti-GFAP (1:250, Lot # 2145934, AB5804, Millipore, MA, USA), overnight at 4°C. After that, they were incubated using FITC-conjugated secondary antibody (1:200, Lot # I1213, sc-2090, Santa Cruz Biotechonology) and counterstained with DAPI (TR-100-FJ, Biosensis, SA, Australia). Finally, the histological sections were mounted onto gelatin-coated slides, and coverslipped with buffered glycerol as a mounting medium.

### Immunohistochemistry Control Reactions

Control reactions for Ten-2 immunohistochemistry were performed by primary and/or secondary antibody omissions. Additionally, an adsorption test was done using Ten-2 primary polyclonal antibody and the control peptide (Ten-2, Lot # E1011, sc-165674P, N-13, 100 μg/0.5 mL, Santa Cruz Biotechnology, CA, USA). For this, Ten-2 antibody (1:100) was incubated with different concentrations of control peptide (1:1; 1:0.1; 1:0.01; 1:0.005) during 24 h in PBS. After that, these solutions were used to incubate histological rat brain sections during 24 h at 4°C, following the procedures adopted in the immunoperoxidase staining method described previously. Similar procedures were performed in a previous study (Torres-da-Silva et al., 2017). All immunolabeled cells noticed in the present study were considered to be “like-immunoreactive” neurons or reactive astrocytes.

### Microscopy and Data Analysis

Histological sections submitted to indirect immunoperoxidase staining method were qualitatively analyzed to identify Ten-2-LI cells using a light microscope (Axiolab A1, Carl Zeiss, BW, Germany) coupled to a digital camera (AxioCam MRc5, Carl Zeiss, BW, Germany). The selected areas were captured using imaging software (Zen2, Carl Zeiss, BW, Germany). When necessary, brightness, contrast and intensity were adjusted in the digital images, without changing the immunolabeling pattern, using Corel Draw software (Corel Corporation, ON, Canada).

Histological sections submitted to double indirect immunofluorescence were quantitatively (GFAP-LI/Ten-2-LI) or qualitatively (GFAP-LI/LPHN-LI) analyzed. For this, the sections were analyzed under a 40 × objective lens and images were captured by a confocal laser scanning microscope (TCS-SP5 AOBS Tandem Scanner, LEICA, HE, Germany) coupled to an inverted optical microscope (Leica DMI 6000CS) from Electron Microscopy Center of the Institute of Biosciences of Botucatu (IBB-UNESP, Botucatu, SP, Brazil). The confocal microscope is equipped with Diode, Helium-Neon and Argon lasers enabling the excitation wavelength lines of 405–633 nm. Fluorochromes were detected sequentially, and we carefully used fluorophores situated far apart in the fluorescence emission spectrum, to avoid a false positive colocalization result. The background marking was controlled in real time through the voltage photomultiplier and adjusted to obtain the best compromise between sensitivity and non-specificity. Planapochromat objectives of 20 × , 40 × and 63 × (numerical aperture 1.30) with oil immersion were used, which allowed a resolution of up to ~150 nm in axes x, y and ~300 nm in the z axis (pinhole of 1 Airy unit). Nuclear astrocyte profiles (DAPI/GFAP-positive), as well as those exhibiting Ten-2-LI were manually quantified using ImageJ software (Rasband, W.S., ImageJ, U. S. National Institutes of Health, Bethesda, Maryland, USA, https://imagej.nih.gov/ij/, 1997-2018.). Five serial histological sections (360 μm equidistant), encompassing the brain lesion or control areas were analyzed in all experimental groups. Next, four microscopic fields (each measuring 15 × 10^4^ μm^2^) flanking the track lesion from each histological section were captured for counting. A blind examiner was previously calibrated for cell counting parameters. The morphology had normal distribution and the data were submitted to ordinary one-way ANOVA, followed by Dunnett's multiple comparison *post-hoc* tests, considering *p* < 0.05 as significant (GraphPad Prism 6, GraphPad Software, Inc., CA, USA).

For 3D-cell reconstructions, twelve reactive astrocytes (Ten-2-LI/GFAP) were analyzed in confocal laser scanning microscope (TCS-SP5 AOBS Tandem Scanner, LEICA, HE, Germany) under a 63 × objective lens and 4 × zoom. The cells were scanned 30–40 times at intervals of 0.3 μm and reconstructed using TCS-SP5 AOBS software (Tandem Scanner, LEICA, HE, Germany).

### RNA Extraction and Conventional RT-PCR

The RT-PCR method was used to check possible Ten-2/TCAP-2 gene expression changes during tissue injury of the cerebral cortex. For this, animals from the experimental groups were anesthetized as previously mentioned, positioned on a stereotaxic apparatus, and the brain surface was again exposed. The cerebral cortex around the lesion was collected under surgical stereomicroscopy (Model MC A-199, DF Vasconcellos, RJ, Brazil), transferred to DNase and RNase free ice-cold saline, and further trimmed to eliminate normal cortex around the lesion to the maximum. This fragment was transferred to appropriate centrifuge tubes with 1.0 mL trizol (#15596026, Life Technologies, CA, USA), immediately homogenized (#985370EUR-04, Tissue-Tearor, Biospec Products, OK, USA) at 30,000 rpm for 40 s and incubated on ice for 5 min. Next, 200 μL of chloroform (#0757, Biochemicals Life Science Research Products, OH, USA) was added, vortexed for 15 s and incubated on ice during 10 min. The tube was submitted to refrigerated (4°C) centrifugation (Mikro 220R, Hettech Zentrifugen, BW, Germany) at 12,000 ×g for 15 min and the upper phase containing the total RNA was transferred to a new tube. After that, 0.5 mL of isopropyl alcohol (#I9030, Sigma-Aldrich, MO, USA) was added to the total RNA solution, incubated at RT for 10 min and centrifuged again at 12,000 ×g, at 4°C for 10 min. The supernatant was discarded and 1 mL of 75 % alcohol (#E7023, Sigma-Aldrich, MO, USA) was added, followed by centrifugation at 7,500 ×g for 5 min at 4°C. Subsequently, the supernatant was discarded and the total RNA pellet was dehydrated at RT for 5–10 min. Total RNA was resuspended in 100 μL of sterilized nuclease-free water (#W4502, Sigma-Aldrich, MO, USA) and heated in dry block (MD-01N, Major Science, CA, USA) for 15 min. To ensure that the total RNA preparation was not contaminated by DNA, the sample was submitted to treatment with Turbo DNA-free Kit (#AM1907, lot 00353291, Life Technologies, CA, USA) following the manufacturer's instructions. The quality and quantity of total RNA of the samples were measured using a spectrophotometer (Optizen POP, Mecasys, South Korea) and submitted to electrophoresis of nucleotides on denaturing gel with 1 % agarose (#N605, Amresco, OH, USA) and 0.0005 % ethidium bromide (#X328, Sigma Aldrich, MO, USA). The remainder of total RNA was stored at −80°C in ultralow freezer (Scientific 923, Forma Scientific, OH, USA).

Previously, all gene expressions were evaluated in order to establish the optimum cycle number in the exponential phase, permiting an accurate semi-quantitative analysis, using RNA samples from control animals. Conventional RT-PCR was used to amplify Ten-2, TCAP-2, GADPH (used for normalization) and neurofilament light (NFL, used for relative data expression) genes (Table 1, *in vivo* analysis). RT-PCR reactions were performed using a commercial kit (#210212, lot 154021850, Qiagen, CA, USA). The total mix (50 μL) of RT-PCR reaction was prepared, containing 10 μL OneStep RT-PCR buffer, 2 μL dNTP mix, 1 μL of each primer (10 nM) (Table 1), 2 μL OneStep RT-PCR enzyme mix, 1–4 μL of total RNA and 26–30 μL RNase-free water. The reaction tubes were placed in a thermal cycler (Mastercycler ProS Eppendorf, GmbH, Germany) for initial reverse transcription for 30 min at 50°C, initial denaturing and PCR activation for 15 min at 95°C and subsequently for 27 cycles (Ten-2 or TCAP-2), 27 cycles (GADPH) or 29 cycles (NFL) of denaturing for 1 min at 94°C, annealing for 1 min at 53°C (Ten-2 or TCAP-2) or 50°C (GADPH; NFL) and elongation for 1 min at 72°C, then for the final elongation cycle for 10 min at 72°C. The DNA samples were stored at 4°C until gel electrophoresis was performed and they were run through a 1.5% agarose gel.

**Table 1:** Primer sets used in RT-PCR and PCR assays.

**Gene**	**Primer Pair**	**Primer sequence (5^**′**^ to 3^**′**^)**	**Exon**	**Product size (base pairs)**
***In vivo*** **RT-PCR ANALYSIS**				
**Teneurin 2**	mTen2e23FWD	5′-gatgtcaactgcatctgctactc-3′	23	491
NM_011856.4	mTen2e23RVS	5′-agtccagtgttcccatcataagtc-3′		
**TCAP-2**	mTCAP2e28FWD	5′-gacaagatgcactacagcatcgag-3′	28	496
NM_011856.4	mTCAP2e28RVS	5′-ccatctcattctgtcttaagaactgg-3′		
**NFL**	rNFLe1FWD	5′-atcagcaacgacctcaagtctatccgc-3	1	761
NM_010910.2	rNFLe1RVS	5′-gagtccagtgttcccatcataagt-3′		
**GADPH**	rGADPHe4FWD	5′-ccatgacaactttggcattg-3	4-6	302
BC023196.2	rGADPHe6RVS	5′-cctgcttcaccttcttg-3′		
***In vitro*** **PCR ANALYSIS**				
**β-actin**	mBetaActine4FWD	5′caggtcatcactattggcaacgag3′	4-6	357
NM_007393.5	mBetaActine6RVS	5′ctcatcgtactcctgcttgctgat3′		
**Teneurin 1**	mTen1e25FWD	5′gtgtcacctgatggcaccctctat3′	25	402
NM_011855.4	mTen1e25RVS	5′tcctgggtatgtcatcaaggccaa3′		
**Teneurin 2**	mTen2e23FWD	5′atcctgaactcgccgtcctcctta3′	23	405
NM_011856.4	mTen2e23RVS	5′ctccaggttctgagtggacacggc3′		
**Teneurin 3**	mTen3e22FWD	5′agtggaatacccggtggggaagcac3′	22	427
NM_011857.3	mTen3e22RVS	5′gtgagtaccgttgatgtcaaagatg3′		
**Teneurin 4**	mTen4e22FWD	5′atcgaccaattcctgctgagcaag3′	22	369
NM_011858.4	mTen4e22RVS	5′catgttctgagtgttcaggaaagg3′		
**TCAP-1**	mTCAP1e32FWD	5′acgtcagtgttgaatgggaggacta3′	32	351
NM_011855.4	mTCAP1e32RVS	5′cctcctgcctatttcactctgtctcat3′		
**TCAP-2**	mTCAP2e28FWD	5′gacaagatgcactacagcatcgag3′	28	496
NM_011856.4	mTCAP2e28RVS	5′ccatctcattctgtcttaagaactgg3′		
**TCAP-3**	mTCAP3e29FWD	5′caacaacgccttctacctggagaac3′	29	506
NM_011857.3	mTCAP3e29RVS	5′cgatctcactttgtcgcaagaact3′		
**TCAP-4**	mTCAP4e29FWD	5′tttgcctccagtggttccatctt3′	29	602
NM_011858.4	mTCAP4e29RVS	5′tggatattgttggcgctgtctgac3′		

### Control Reactions

Control reactions were performed without RNA addition for RT-PCR or with RNA addition for PCR assays (#C1141, GoTaq Flexi DNA Polymerase, Promega, WI, USA), using at least 30–35 cycles in both assays.

### Data Analysis

RT-PCR bands were captured and digitalized under UV light using ImageQuant LAS 500 (GE Healthcare Bio-Sciences AB, Uppsala, Sweden). The optical density of the bands for Ten-2, TCAP-2, GADPH and NFL gene expressions were analyzed by densitometry using ImageQuant LAS 500 (GE Healthcare Bio-Sciences AB, Uppsala, Sweden) software. Absolute data were normalized with GADPH and expressed in relation to NFL gene expression. Ten-2 data were expressed in relation to NFL gene expression, as Ten-2 is present in cortical neurons in control animals and in neurons and astrocytes in animals with cerebral cortex injury. This analysis is more confident because we collected samples more restricted to the injury area, reducing the healthy tissue around it to the maximum, where NFL expression is more elevated due to presence of more neurons. Thus, we can infer that if there is some increase in the Ten-2 gene expression in samples from animals with cerebral cortex injury, this expression increase most likely comes from Ten-2 reactive astrocyte gene expression than from neurons, since NFL expression is low in these animals group.

The gene expression data had normal distribution and were submitted to ordinary one-way ANOVA, followed by Dunnett's multiple comparison *post-hoc* tests, considering *p* < 0.05 as significant (GraphPad Prism 6, GraphPad Software, Inc., CA, USA).

### *In vitro* Study

#### Cell Culture

We searched for an astrocyte cell lineage that expresses teneurin gene expression in order to adopt it in future *in vitro* assays. For this, C8D1A mouse cerebellar immortalized astrocytes (#CRL-2541, ATCC, VA, USA) were used. In addition, based on the fact that TCAP-1 is a bioactive peptide in neurons supported by *in vitro* and *in vivo* studies, we tested whether TCAP-1 is able to change calcium signaling in this cell lineage.

This astrocytes cell lineage was cultured in Dulbecco's Modified Eagle's Medium (DMEM) containing 4,500 mg/ L glucose content, 4 mM L-glutamine, 1 mM sodium pyruvate, 1,500 mg/ L sodium bicarbonate (#30-2002, ATCC, VA, USA) with 10% fetal bovine serum (FBS, #12483020, Thermo Fisher Scientific, MA, USA), 100 μg/ mL penicillin and 100 μg/ mL streptomycin (#15140-122, Thermo Fisher Scientific, VA, USA) added to the medium. Astrocytes were incubated at 37°C, 5% CO_2_ and cells were maintained at 70–80% confluency.

### RNA Extraction, Reverse-Transcription, and Polymerase Chain Reaction (PCR)

Once at 70–80% confluency in a 6-well plate, C8D1A astrocytes were serum-starved for 3 h. Then, 1 mL trizol reagent was added to each well to extract RNA from the cells after which they were incubated for 2 min at RT. Lysates were transferred and 200 μL of chloroform (#C3300, ACP Chemicals, QC, Canada) was added. The solution was thoroughly mixed and incubated at RT for 3 min. Subsequently, the samples were centrifuged at 12,000 ×g for 15 min at 4°C. The RNA-containing supernatant was transferred to a new tube and 500 μL of isopropanol (#A426, Thermo Fisher Scientific, MA, USA) was added. The solution was incubated at RT for 10 min and then centrifuged at 14,000 ×g for 10 min and 4°C. After centrifugation, the supernatant was discarded and the pellet was washed using 75% ethanol (EtOH) (#PO16EA95, Commercial Alcohols, Canada) and centrifuged at 7,400 ×g for 5 min. After two rounds of EtOH washes and centrifugation, the EtOH was removed and the pellet was re-suspended in 20 μL diethyl pyrocarbonate (DEPC)-water (#W4502, Sigma-Aldrich, MO, USA). RNA sample absorbance was determined using the NanoDrop 2000 spectrophotometer at 260 nm and 280 nm wavelengths (Thermo fisher scientific, MA, USA, v.1.4.2).

The RNA extracted from C8D1A immortalized astrocytes was reverse-transcribed to complementary DNA (cDNA). RNA-free H_2_O, random primers (#SO142, Thermo Fisher Scientific, MA, USA) and dNTPs (#RO192, Thermo Fisher Scientific, MA, USA) were added to the sample RNA and the sample was heated to 65°C for 5 min. The sample was left on ice for 1 min, after which 5X first-strand buffer (#YO2321, Invitrogen, CA, USA) and 0.1 M DTT (#YO0147, Invitrogen, CA, USA) were added, mixed and left at RT for 2 min. Subsequently, Superscript II RT (#100004925, Invitrogen, CA, USA) was added, after which the sample was left at RT for 10 min, heated to 42°C for 50 min and then heated to 70°C for 15 min.

To perform PCR, mastermix containing ddH_2_O, Taq buffer (#B33, Thermo Fisher Scientific, MA, USA), MgCl_2_ (#B34, Thermo Fisher Scientific, MA, USA) and dNTPs was prepared and appropriate primer pairs were added (Table 1, *in vitro* analysis) along with the cDNA template and Taq polymerase (#EP0402, Thermo Fisher Scientific, MA, USA). The reaction tubes were then placed in the thermal cycler (#6331000025, Eppendorf, Germany) for the first cycle of denaturing for 7 min at 95°C, subsequently for 35 repeated cycles of denaturing for 1 min at 95°C, annealing for 1 min 30 s at 60–65°C and elongation for 35 s at 72°C and then for one final cycle of elongation for 5 min at 72°C. The DNA samples were stored at 4°C until gel electrophoresis was performed and they were run through a 3% agarose gel (#9012-36-6, BioShop, Canada) and imaged using Image-Lab software (Bio-Rad, CA, USA, v.4.1).

### Intracellular Calcium Fluorescence Microscopy

Cells were grown on coverslips and once at 60% confluency in a 6-well plate, they were incubated for 30 min at 37°C in 3.6 μM Fluo-4 acetomethyl (AM) ester (1 μg/μL dissolved in DMSO; #F14201, Thermo Fisher Scientific, MA, USA) in culture medium. Subsequently, the cells were washed with artificial cerebrospinal fluid (aCSF) (135 mM NaCl, 4.5 mM KCl, 2 mM MgCl_2_6H_2_O mM, 10 mM HEPES, 10 mM glucose, 2 mM CaCl_2_2H_2_O) and the coverslip remained in aCSF until it was mounted to the stage of an inverted fluorescence microscope (Zeiss Axio Observer.Z1, Carl Zeiss Microimaging, BW, Germany) for perfusion. The microscope was equipped with a 40 × oil immersion lens (1.4 NA, Carl Zeiss Microimaging, BW, Germany) and a digital CCD camera (C4742-80, Hamamatsu Photonics, Hamamatsu, Japan) to capture fluorescent images. Velocity cellular imaging software (Improvision, version 4.3.2) was used to obtain fluorescent images. Solutions were administered using a perfusion system and teneurin C-terminal associated peptide (TCAP-1) was added to aCSF for treatment at a 100 nM concentration.

### Data Analysis

Data from calcium assays were normalized to the fluorescence ratio baseline and presented as standard error of the mean (SEM). Data exhibited normal distribution and were submitted to comparisons of multiple conditions using two-way ANOVA with a Bonferroni *post hoc* test, considering *p* < 0.05 as significant (v.5 GraphPad Prism 6, GraphPad Software, Inc., CA, USA).

## Results

### *In vivo* Study

#### Immunoperoxidase Staining

**Figure 1:**
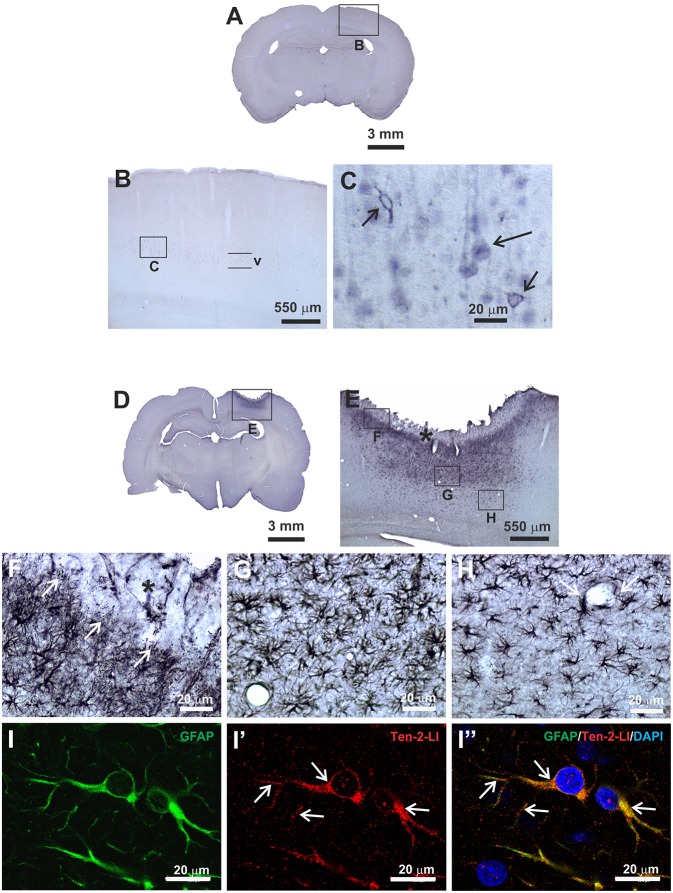
Immunoperoxidase staining **(A–H)** and double indirect immunofluorescence **(I–I”)** methods to analyse Ten-2 immunolabeling pattern in histological section of adult rat cerebral cortex. **(A–C)**, control group, observe neurons in cerebral cortex layer V exhibiting discrete immunoreactivity to Ten-2, homogenously distributed in cell cytosol (large arrow) or associated with cell membrane (small arrows). In **(D–H)**, mechanical brain lesion in cerebral cortex from 48 h postoperative period group, showing strong immunoreactivity to Ten-2 in reactive astrocytes. In **(E,F)**, note Ten-2-LI reactive astrocytes with palisading aspect sending some cell extensions (arrows) to haemorraghic area (**F**, black asterisk). Ten-2-LI hipertrophic reactive astrocytes are densely grouped immediately below **(G)** and decreasing toward deeper layers of the cerebral cortex **(H)**. Reactive astrocytes exhibited large cell bodies with numerous long arborized cell extensions **(G,H)**, sometimes encircling the blood vessel (**H**, arrow). In **(I–I”)** immunolabeling pattern of Ten-2 in reactive astrocytes (GFAP-LI) analyzed by confocal microscopy. Observe that immunolabeling to Ten-2 is distributed in the cytosol of the cell body and cell extensions (arrows), exhibiting a punctiform pattern and sometimes associated with plasmatic membrane. GFAP-LI, glial fibrillary acidic protein-like immunoreactive; Ten-2-LI, teneurin-2-like immunoreactive; V, cerebral cortex layer V.

The immunoperoxidase staining method was important to detail the morphological characteristics of neural cells that exhibited immunoreactivity to Ten-2. Ten-2-like immunoreactive (Ten-2-LI) cells in the cerebral cortex from control animals were represented mainly by pyramidal neurons situated in layer V. However, this immunolabeling was discrete and exhibited two patterns, one associated with the cell membrane delineating the cell body (perikarya) and the other one homogenously distributed in the cell cytoplasm of the cell body and apical dendrite (Figures 1A-C). In animals with mechanical brain injury, Ten-2-LI cells significantly changed in the cerebral cortex with strong immunolabeling in reactive astrocytes in all postoperative groups (Figures 1D–H). Occasionally, rare Ten-2-LI neurons scattered among reactive astrocytes were observed. The normal cortical areas around the brain lesion maintained the same immunolabeling pattern in neurons.

**Figure 2:**
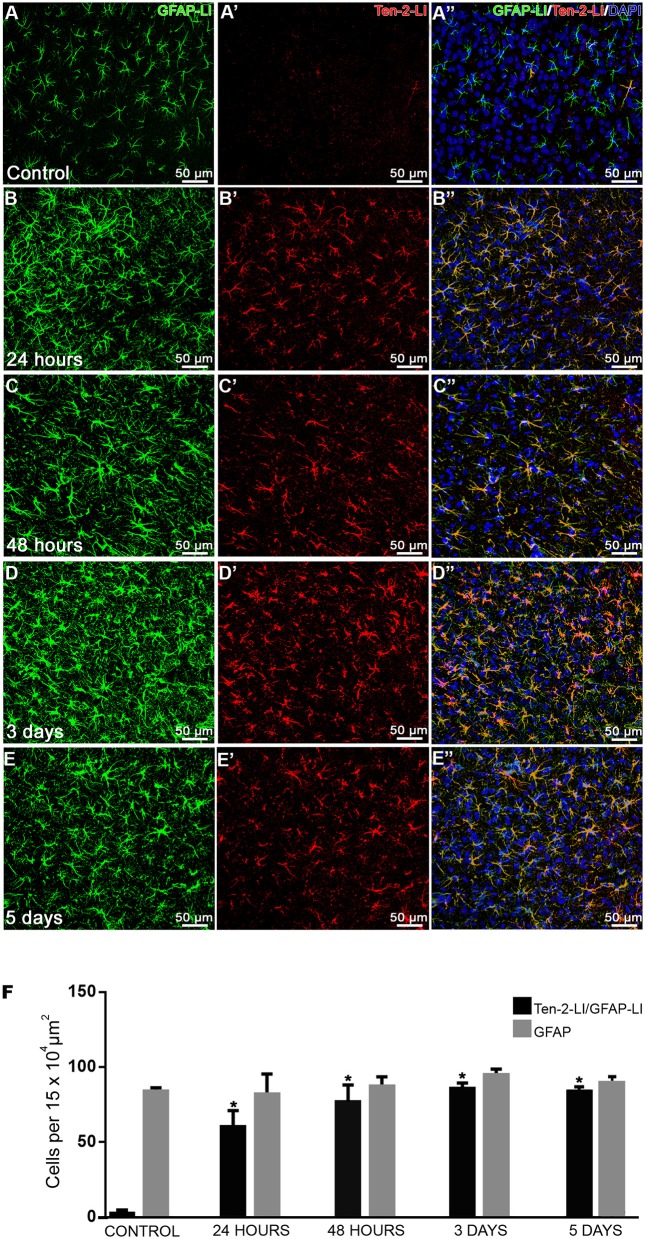
Temporal analysis of Ten-2-LI reactive astrocytes. **(A–E”)** confocal microscopy photomicrographs showing double immunolabeling in reactive astrocytes (GFAP-LI/ Ten-2-LI) with nuclear DAPI staining in cerebral cortex from control, 24 h, 48 h, 3 and 5-day groups. Note that these reactive astrocytes are clearly visible at 24 h **(B–B”)** and nearly all of them exhibit immunoreactivity to Ten-2 in all experimental groups. **(F)** quantitative analysis of absolute values (mean ± SEM) of astrocyte cell profiles exhibiting immunoreactivity to Ten-2 (GFAP-LI/Ten-2-LI) in all experimental groups (*n* = 5, five animals per group). Note that reactive astrocyte cell profiles (GFAP-LI) showed no statistical difference among all groups, indicating that there is no cell proliferation. However, note that reactive astrocyte cell profiles (GFAP-LI/Ten-2-LI) significantly increased (*p* < 0.0001) in all experimental groups with mechanical brain injury compared to control group. Mean (± SEM) values from each group were submitted to one-way ANOVA and Dunnett's multiple comparisons *post hoc* test, considering *p* < 0.05 as significant. ^*^ Statistically significant difference when compared with control group (*p* < 0.0001). GFAP-LI, glial fibrillary acidic protein-like immunoreactive; Ten-2-LI, teneurin-2-like immunoreactive.

Frequently, reactive astrocytes were nearly absent in the area closest to the needle track or haemorragic area. Close to that area, only Ten-2-LI palisading reactive astrocytes sending cell processes to haemorragic area were observed, mainly in later postoperative periods (Figures 1E,F). Adjacent to that area, Ten-2-LI reactive astrocytes showed significant hypertrophy, decreasing toward deeper cerebral cortex layers and white matter (Figures 1E,G,H, 2). At times, Ten-2-LI reactive astrocytes showed clear cell extensions projecting to blood vessels (Figure 1H).

Control reactions for Ten-2 immunohistochemistry by pre-adsorption Ten-2 epitope resulted in complete absence of immunolabeling in neurons and reactive astrocytes in all peptide concentrations (Figure S1). In addition, no immunolabeling was noticed after primary and/or secondary antibody omissions (Figure S1).

For comparative analysis, additional data concerning indirect immunoperoxidase staining for detection of Ten-1, Ten-3, or Ten-4 in the cerebral cortex of animals with mechanical brain injury of the cerebral cortex are also presented (Figure S2). There is no immunolabeling for these proteins in reactive astrocytes. Only Ten-1-LI cortical neurons were evident, but it was quite similar in relation to the control groups (Figure S2).

### Double Immunofluorescence

Since the main cells that exhibited immunoreactivity to Ten-2 were reactive astrocytes during tissue injury, the double immunofluorescence method was used in order to count nuclear astrocyte profiles (nuclear DAPI staining/GFAP-LI astrocytes) exhibiting immunoreactivity to Ten-2 in the experimental groups. GFAP-LI astrocytes showed homogenous distribution in the cerebral cortex of the control group. Immunoreactivity was present in the cytosol of the cell body and in its thin cell extensions (Figures 1I-I”; Figure S3; Video S1). Astrocytes rarely exhibited detectable immunolabeling to Ten-2 in control group animals (Figures 2A–A”). On the other hand, animals with brain injury from all postoperative periods showed a clear and strong Ten-2 immunolabeling in reactive astrocyte cell profiles (Figures 2B–E”). These cells exhibited large cell bodies, besides arborized and elongated cell extensions (Figures 2B-E”; Figure S3; Video S1). Interestingly, the immunolabeling pattern to Ten-2 was punctiform and distributed in the cytosol of reactive astrocytes (Figures 1I-I”; Figure S3; Video S1).

Quantitative analysis demonstrated that the number of nuclear astrocyte cell profiles DAPI/GFAP-LI) did not show statistical difference among all experimental groups (Figure 2F). However, the number of Ten-2-LI nuclear astrocyte profiles (DAPI/GFAP-LI/Ten-2-LI) was significantly increased in animals submitted to mechanical brain injury in all postoperative periods (*p* < 0.0001), compared to the control group (Figure 2F).

In order to investigate with more detail whether the immunoreactivity to Ten-2 was present in the cell membrane and/or in inner parts of the reactive astrocytes, some histological sections with Ten-2-LI reactive astrocytes (DAPI/GFAP-LI/Ten-2-LI) were used for 3D reconstruction in confocal microscope (Figure S3; Video S1). The cells clearly showed that the immunolabeling was present in the cytoplasm with granular arrangement or sparce punctiform labeling linked to the cell membrane (Figure S3; Video S1).

**Figure 3:**
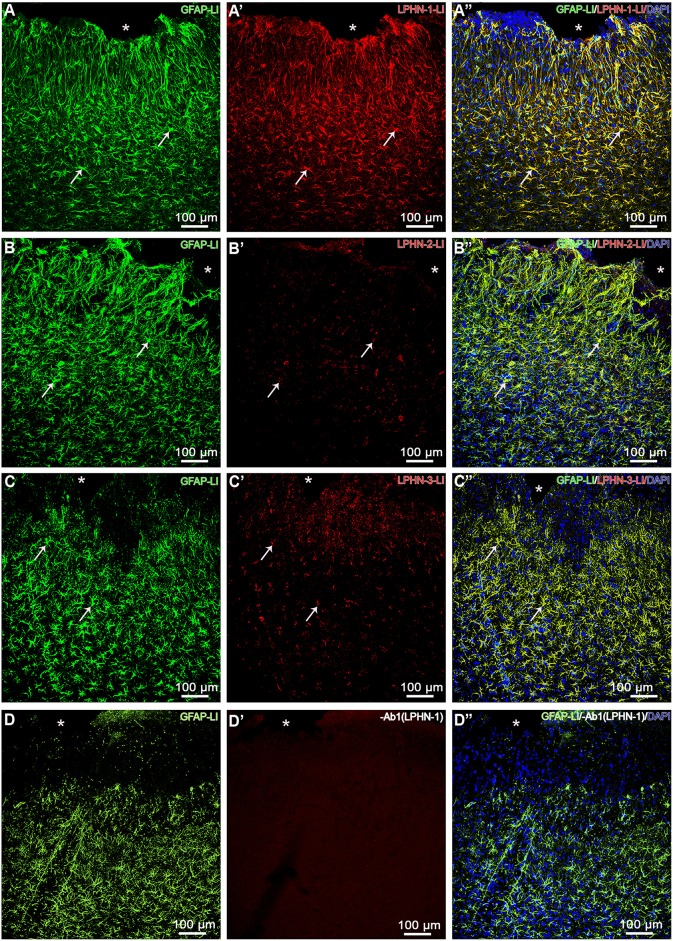
LPHN-LI reactive astrocytes. Confocal microscopy photomicrographs showing reactive astrocytes (GFAP-LI) stained with nuclear DAPI and exhibiting immunoreactivity to LPHN-1 **(A–A”)**, LPHN-2 **(B–B”)**, and LPHN-3 **(C–C”)** in cerebral cortex with mechanical brain lesion in cerebral cortex from 3-day postoperative period group. Note that nearly all reactive astrocytes exhibited substantial immunoreactivity to LPHN-1 **(A-A”)**, moderate to LPHN-3 **(C–C”)** and modest to LPHN-2 **(B–B”)** (arrows). Ab1 (LPHN-1), omission of LPHN-1 primary polyclonal antibody; GFAP-LI, glial fibrillary acidic protein-like immunoreactive; LPHN-1-LI, latrophilin-1-like immunoreactive; LPHN-2-LI, latrophilin-2-like immunoreactive; LPHN-3-LI, latrophilin-3-like immunoreactive; ^*^, cortical lesion area.

Based on the fact that latrophilins are involved in heterophilic interactions with teneurins, we submitted histological sections for double immunofluorescence to qualitatively evaluate whether immunoreactivity to LPHN is evident in reactive astrocytes (GFAP-LI). Animals with mechanical brain injury exhibited strong immunoreactivity to LPHN-1 in reactive astrocytes, moderate to LPHN-3 and discreet to LPHN-2 (Figure 3). Sections from control animals did not show any LPHN-LI astrocytes. Control reactions with primary antibody omission for latrophilins resulted in absence of immunolabeling in reactive astrocytes (Figure 3).

### Conventional RT-PCR

**Figure 4:**
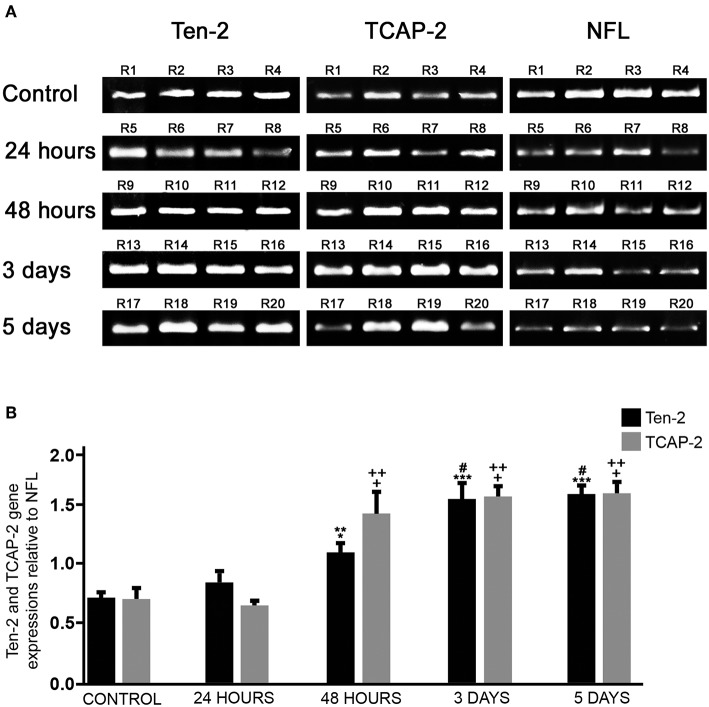
Ten-2/TCAP-2 genic expression. **(A)** conventional RT-PCR products using total RNA extracted from cerebral cortex of all experimental groups (*n* = 4, four animals per experimental group) and Ten-2, TCAP-2 and neurofilament light (NFL) primers, visualized in 1.5% agarose gel stained with bromide ethidium. **(B)** Semi-quantitative analysis of relative gene expression (mean ± SEM) of Ten-2 and TCAP-2 bands normalized in relation to GADPH and expressed in relation to NFL. Mean (± SEM) values from each group were analyzed by one-way ANOVA and Dunnett's multiple comparisons *post hoc* test, considering *p* < 0.05 as significant. TCAP-2: + statistically significant difference when compared with control group (*p* < 0.0001); ++ statistically significant difference when compared with 24 h group (*p* < 0.0001). Ten-2: ^*^statistically significant difference when compared with 24 h group (*p* < 0.05) ^**^statistically significant difference when compared with control group (*p* < 0.001); ^***^statistically significant difference when compared with control group (*p* < 0.0001); # statistically significant difference when compared with 24 h group (*p* < 0.0001). R1-R20, animal number identification.

RT-PCR analysis was used in order to confirm Ten-2 and TCAP-2 expression as well as to check possible changes during mechanical cerebral cortex injury. RT-PCR analysis showed both Ten-2 and TCAP-2 mRNA expression in all experimental groups (Figure 4). Mechanical brain-injured groups (48 h, 3 and 5 days) revealed a significant increase in Ten-2 and TCAP-2 mRNA expressions, compared with control (*p* < 0.0001) and 24 h (*p* < 0.0001) groups (Figure 4). Control reactions in PCR using RNA samples and Ten-2, TCAP-2 or NFL primers did not show any bands (Figure S4). Similarly, RT-PCR without addition of RNA samples showed no bands.

### *In vitro* Study

#### Conventional RT-PCR

**Figure 5:**
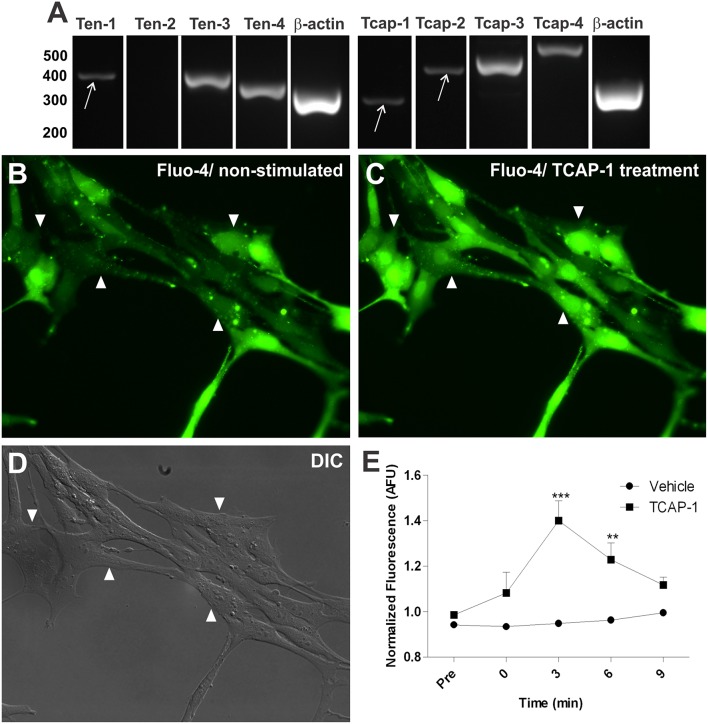
*In vitro* characterization of Ten/TCAP gene expressions and TCAP-1 induced activation in mouse cerebellar astrocytes. Gene expression was determined using RT-PCR with RNA extracted from C8D1A mouse cerebellar astrocytes. **(A)** teneurins 1, 3 and 4 were expressed (*n* = 4) and teneurin 2 was not expressed (*n* = 4). TCAPs 1-4 were expressed (TCAP-1, *n* = 3; TCAP-2, *n* = 5; TCAP-3, *n* = 3; TCAP-4, *n* = 3). β-actin served as positive control (*n* = 5). **(B)** fluo-4 fluorescence shown prior to 100 nM TCAP-1 administration (non-stimulated). **(C)** fluo-4 fluorescence shown 3 min after 100 nM TCAP-1 administration (TCAP-1 treatment). **(D)** differential interference contrast image (DIC) C8D1A astrocytes. Arrows are showing discreet band. **(E)** normalized fluo-4 fluorescence comparing vehicle (aCSF) and 100 nM TCAP-1 treated cells (*n* = 3 for each treatment, where each n is an average of five cells per coverslip). Mean (± SEM) values from each group were submitted to two-way ANOVA and Bonferroni *post hoc* test. ^**^p < 0.01; ^***^p < 0.001.

We searched for teneurin and TCAP gene expression in the C8D1A mouse cerebellar immortalized astrocytes, in order to evaluate whether these proteins are expressed in cell lineage astrocytes which can be used in future assays. RT-PCR analysis showed immortalized cerebellar astrocytes expressing Ten-1, Ten-3, and Ten-4, but not Ten-2 (Figure 5A). Gene expression of TCAP-1-4 was also present in the immortalized astrocytes (Figure 5A).

### Intracellular Calcium Fluorescence Microscopy

Based on the fact that TCAP-1 is a bioactive peptide in neurons supported by *in vitro* and *in vivo* studies (Al Chawaf et al., 2007a,b; Trubiani et al., 2007; Tan et al., 2009, 2011; Chand et al., 2012), we tested whether TCAP-1 is able to change calcium signaling in immortalized astrocytes. Thus, immortalized cerebellar astrocytes were treated with synthetic TCAP-1. These cells exhibited substantial increases in intracellular Ca^2+^, as quantified by the emitted fluorescence of Fluo-4-AM ester binding to Ca^2+^, after TCAP-1 treatment (Figures 5B–D). The level of intracellular Fluo-4-AM fluorescence increased significantly at 3 (*p* < 0.001) and 6 (*p* < 0.01) minutes after TCAP-1 treatment (100 nM), in relation to vehicle treatment (Figure 5E).

## Discussion

The present study is the first to show that Ten-2 and TCAP-2 are upregulated in reactive astrocytes after mechanical brain injury induced by needle-insertion lesion in the cerebral cortex of adult rats. LPHN-1, the main endogenous receptor for a Ten-2 splice variant named Lasso, was also evidenced in reactive astrocytes by the immunohistochemistry method. *In vitro* analysis showed that immortalized cerebellar astrocytes also express additional Ten/TCAP homologs and increase calcium uptake after TCAP-1 treatment. These findings disclose a new functional role of the Ten/TCAP system in glia during CNS tissue repair.

Several studies have reported that reactive astrocytes secrete several anti-inflammatory substances, as well as act as a barrier for harmful substances that diffuse from blood vessels (Sofroniew, 2015). Considering gene expression analysis in brain injuries, a previous study showed that reactive astrocytes collected from different regions of the mouse brain with middle cerebral artery occlusion (MCAO) had significant gene up-regulations, where Ten-2 gene was one of the 50 most up-regulated genes (Zamanian et al., 2012). Furthermore, the Ten-2 gene was up-regulated in the human frontal cortex from patients with amyotrophic lateral sclerosis (Andrés-Benito et al., 2017), as well as in patients with chronic traumatic encephalopathy (Seo et al., 2017). There is a rare astrocyte disease caused by dominant gain-of-function mutations in the GFAP gene named Alexander disease (Messing et al., 2012). Astrocytes differentiated from induced pluripotent stem cell (iPSCs) samples collected from patients developing Alexander disease revealed that ODZ2 (Ten-2) is one of the most up-regulated genes in comparison with astrocytes differentiated from healthy subjects (Kondo et al., 2016). These experimental and clinical data support a possible participation of Ten-2 in brain disorders, mainly in astrocytes under certain CNS diseases. Our *in vivo* immunohistochemistry data strongly suggest that reactive astrocytes are synthetizing Ten-2 and its up-regulation was confirmed by significant increase of Ten-2 mRNA in the cerebral cortex of animals with mechanical brain injury. Supporting this statement, we did not notice an increase of immunoreactivity to Ten-2 in neurons that produce Ten-2, or in microglia, or oligodendrocyte in the cerebral cortex with mechanical brain injury. Moreover, it is important to mention that reactive astrocytes are not phagocytes like microglia, which could endocyte possible Ten-2 released from neurons. Based on these considerations, it can be concluded that immunoreactivity to Ten-2 evidented in reactive astrocytes is from the Ten-2 gene expression upregulation in these cells.

It is worth mentioning that we adopted some controls for our immunohistochemistry analysis. Omission of the primary antibodies for Ten-2 and LPHN and/or secondary antibody resulted in absence of immunolabeling. Pre-adsorption test for Ten-2 eliminated any immunolabeling of neurons and reactive astrocytes suggesting that polyclonal antibody used in the present study was specific to Ten-2 epitope. However, we did not perform immunohistochemistry assay controls using our primary antibody lots or aliquotes in histological brain sections or in cell lineages generated by knockout procedures for teneurins, latrophilins or GFAP. It is known that one of the best controls to confirm antibody specificity is through immunohistcohemical assays using samples from knockout models (Saper and Sawchenko, 2003; Burry, 2011). Based on these considerations and limitations of the present study, we have adopted the term “like-immunoreactive” for all immunolabeling detected in the neurons and/or reactive astrocytes.

Brain injury has been used as a model to study CNS repair. The needle-insertion lesion model is a focal and controlled injury of the rat cerebral cortex, which exhibits two different lesion areas (Purushothuman et al., 2013; Purushothuman and Stone, 2015). One area is along the track of the needle insertion, where there is mechanical tissue disruption with neuronal and synapse degeneration, associated with haemorrhagic and several extracellular modifications (Purushothuman et al., 2013). The other area is tissue flanking the track, where there is non-significant neuronal degeneration and intracellular transient effects, up-regulating different mechanisms for self-protection to minimize the effects of injury (Purushothuman et al., 2013; Purushothuman and Stone, 2015), similar to the tissue surrounding intracerebral haemorrhagic area. In the present study, only cell extensions from Ten-2-LI reactive astrocytes were noticed close to the haemorraghic area induced by needle insertion; while in the flanking area, nearly all GFAP astrocyte profiles exhibited immunoreactivity to Ten-2. Thus, Ten-2/TCAP-2 reactive astrocytes are mainly located in an area that expresses neuroprotective molecules. Interestingly, TCAP-1 acts as a neuroprotective molecule in immortalized hypothalamic neurons, as it induced superoxide dismutase, superoxide dismutase-1 copper chaperone and catalase enzymes (Trubiani et al., 2007).

CNS injuries or diseases implicate several neural and non-neural cell types to protect the brain and try to recover its function (Burda and Sofroniew, 2014). Astrocytes are a pivotal cell type involved in brain damage, including stroke, tumors, infections, neurodegeneration, traumatic brain injury, chemical toxicity and epilepsy (Sofroniew and Vinters, 2010; Kang and Hébert, 2011; Liu and Chopp, 2016). After the occurrence of brain injury, the astrocytes become active, undergo hypertrophy and hyperplasia processes (astrogliosis), and consequently up regulate GFAP, vimentin and other mediators (Eng and Ghirnikar, 1994; Sofroniew and Vinters, 2010; Burda and Sofroniew, 2014; Liu et al., 2014; Sofroniew, 2015; Burda et al., 2016; Liu and Chopp, 2016). In the present study, it was observed that reactive astrocytes exhibited significant immunoreactivity to Ten-2 up to 5 days after injury. After that, the expression decreased significantly up to 30 days postoperatively (data not shown). Thus, the presence of Ten-2/TCAP-2 in astrocytes seems significant only on the first days after cortical injury, indicating its involvement during the cascate of events to minimize the inflammatory mechanism, as evidented in astrocytes of subtype A2 (Liddelow and Barres, 2017).

The *in vitro* analysis using immortalized mouse cerebellar astrocytes was useful; as it confirmed that astrocytes also express Ten/TCAP homolog mRNAs, supporting their application in further studies to detail the possible functions of these proteins and their signaling mechanism. In contrast with our *in vivo* data, immortalized cerebellar astrocytes expressed additional Ten/TCAP homologs. However, this differential gene expression can be due to the *in vitro* environment, which does not simulate all *in vivo* conditions. Moreover, this cell lineage is derived from a newborn mouse cerebellum (postnatal day 8), which has particular astrocytes, such as Bergmann glia and velate astrocytes. It is interesting to mention that there are a few studies analyzing Ten-2 distribution in the cerebellum during development or in adult rodent brain and they did not mention Ten-2 presence in astrocytes (Otaki and Firestein, 1999; Zhou et al., 2003). One study only pointed out noticeable Ten-2 presence in neurons situated in molecular and Purkinge layer cells and less pronounced presence in the granular layer in the mouse cerebellum (Zhou et al., 2003); while Ten-2 presence was not noticed in the cerebellum in adult Sprague-Dawley rats (Otaki and Firestein, 1999).

Teneurins are a family of proteins mainly expressed in the CNS during development and recent studies have shown that they stablish homophilic or heterophilic interactions (Silva et al., 2011; Mosca, 2015; Woelfle et al., 2015). Heterophilic interactions between teneurins with integrins, dystroglycans and latrophilins have been suggested by several studies (Trzebiatowska et al., 2008; Silva et al., 2011; Topf and Chiquet-Ehrismann, 2011; Boucard et al., 2014; Mosca, 2015; Woelfle et al., 2015; Vysokov et al., 2016; Li et al., 2018). Integrins and dystroglycans are present in astrocytes and play a role of maintaining blood-brain barrier (BBB) homeostasis (Guadagno and Moukhles, 2004; del Zoppo and Milner, 2006; Wolburg-Buchholz et al., 2009; Sofroniew and Vinters, 2010). A possible interaction between integrins and/or dystroglycans with Ten-2/TCAP-2 in some reactive astrocytes related to BBB components can be considered, since our immunohistochemistry analysis revealed several Ten-2-LI reactive astrocytes projecting cell extensions to the vicinity of the blood vessels. Supporting this assumption, there are some studies indicating teneurin interaction with integrins and distroglycan in neurons as well as in other tissues like connective tissue and testis in other tissues (Löer et al., 2008; Trzebiatowska et al., 2008; Topf and Chiquet-Ehrismann, 2011; Chand et al., 2012, 2014).

LPHN-1 is the main endogenous receptor for a Ten-2 splice variant named Lasso. Remarkably, substantial immunoreactivity to LPHN-1, moderate to LPHN-3 and modest to LPHN-2 was observed in reactive astrocytes in the present study. Possibly, reactive astrocytes display Ten-2 and its receptor LPHN-1 during brain injury. This information indicates that Ten-2 and/or its related proteins can be released by reactive astrocytes, inducing self-stimulation or self-inhibition by coupling in the LPHN-1 autoreceptor and modulating the intracellular signaling mechanism. In addition, the possibility that reactive astrocytes simultaneously exhibit Ten-2 and LPHN-1 can also indicate that these cells stablish an intercellular interaction by heterophilic coupling involving these proteins. A previous study showed that LPHN-1 and Ten-2 are sited in distinct parts of the synapse, where the former is in the presynaptic and the latter in the postsynaptic membrane of neurons (Boucard et al., 2014). Lasso interacts with LPHN-1 in neurons, inducing calcium signaling in these cells (Silva et al., 2011; Boucard et al., 2014; Mosca, 2015; Woelfle et al., 2015; Vysokov et al., 2016). It is known that calcium signaling in astrocytes results in gliotransmitter release, modulating neurons through tripartite synapses (Araque et al., 2014; Gundersen et al., 2015; Mitterauer, 2015; Covelo and Araque, 2016). Tripartite synapses are formed by pre- and post-synaptic neuronal membranes, besides cell extensions from nearby astrocytes, permitting cross-interactions and modulation between astrocytes and neurons (Araque et al., 2014; Gundersen et al., 2015; Covelo and Araque, 2016). In addition, astrocytes establish ionic coupling through gap junctions, particularly involving calcium, which allows for the synchronization of their activity; thus, enabling several astrocytes to act as a syncytium (Araque et al., 2014; Gundersen et al., 2015; Covelo and Araque, 2016). This characteristic enables a small number of activated astrocytes to substantially expand their ability to modulate neuronal activity in larger areas of the CNS (Covelo and Araque, 2016). Therefore, a possible interaction among Ten-2-related proteins and LPHN in reactive astrocytes could induce calcium uptake and modulate gliotransmitter release by these cells or eventually modulate their vascular functions. In line with this assumption, our *in vitro* assays using immortalized astrocytes demonstrated that TCAP-1 treatment was able to induce significant calcium signaling in these cells. Such data support the idea that the carboxy terminus of Ten-2 plays a fundamental role in calcium signaling in neurons (Silva et al., 2011; Boucard et al., 2014; Mosca, 2015; Woelfle et al., 2015; Vysokov et al., 2016) and TCAP is possibly part of this mechanism in neurons and astrocytes.

Teneurins may be cleaved at the extracellular domain, specifically in the C-terminal region, resulting in TCAP, a bioactive peptide (Qian et al., 2004; Wang et al., 2005; Lovejoy et al., 2006). TCAP-1 can also be separately encoded by the last exon of Ten-1 (Chand et al., 2013). TCAP acts in stress modulation, neuroprotection, CRF-induced cocaine addiction reinstatement, among other functions (Qian et al., 2004; Wang et al., 2005; Lovejoy et al., 2006; Al Chawaf et al., 2007a,b; Trubiani et al., 2007; Kupferschmidt et al., 2011; Tan et al., 2011; Chen et al., 2013; Erb et al., 2014; Colacci et al., 2015). In the present study, it was observed that TCAP-2 expression was increased in the injured cerebral cortex; however, there is no available antibody to analyze TCAP-2 immunoreactivity. Thus, it can only be hypothesized that TCAP-2 is present in reactive astrocytes, corroborated by TCAP-2 gene expression data from *in vitro* analysis.

Finally, reactive astrocytes showed immunolabeling to Ten-2 present in the cytoplasm with granular arrangement or linked to the cell membrane with disperse punctiform distribution. These data can suggest that Ten-2/TCAP-2 proteins in reactive astrocytes may not work only as cell interaction molecules, since the immunolabeling did not show a typical plasmatic membrane presence in these cells. The possibility that Ten-2/TCAP-2 proteins are released by a secretory regulated pathway, generating soluble proteins exerting other functional roles has been suggested in previous studies (Chand et al., 2013; Vysokov et al., 2016). Moreover, the Ten-2 punctiform immunolabeling visible in the cell membrane from reactive astrocytes may characterize a specific membrane domain enriched with Ten-2 transmembrane proteins or some cell membrane regions with docked secretory granules filled with Ten-2/TCAP-2 proteins. Further studies analyzing Ten-2-LI reactive astrocytes by immunohistochemistry combined with electron microscopy could help elucidate this question. Unfortunately, there are no commercially available antibodies raised for different parts of the Ten-2 that could clarify whether Ten-2 upregulation has the role to produce Ten-2 transmembrane protein and/or secretory related proteins for autocrine or paracrine actions.

## Conclusions

Based on the limitations of the present study, a significant increase in Ten-2-LI reactive astrocytes was demonstrated for the first time after mechanical injury of the adult rat cerebral cortex. Reactive astrocytes also exhibited immunoreactivity to LPHN-1, the main endogenous receptor of Ten-2 splice variant named Lasso. Ten-2/TCAP-2 gene expression was also up-regulated in the cerebral cortex with mechanical brain injury. *In vitro* analysis using immortalized cerebellar astrocytes confirmed that these neural cells are potentially able to express additional Ten/TCAP homologs and that TCAP-1 treatment significantly increased calcium signaling in this cell line. Further studies are necessary to evaluate the role of Ten-2/TCAP-2 in reactive astrocytes, as well as to investigate the potential maneuver of these proteins as adjuvant therapies in CNS injury repair.

## Ethics Statement

The experimental protocols for animal handling and care were approved by the Institutional Committee of Animal Welfare (CEUA, process number 2015-00318) from School of Dentistry of Araçatuba (UNESP, Araçatuba, SP, Brazil).

## Author Contributions

GT: *in vivo* study: surgery procedures, immunohistochemistry and analysis, RT-PCR and analysis, manuscript drafting. OM: *in vitro* study: culture cells, RT-PCR and analysis, calcium signaling and analysis, manuscript drafting. KT: *in vivo* study: animal handling, immunohistochemistry and analysis, RT-PCR, manuscript drafting. AD: *in vivo* study: immunohistochemistry and analysis, manuscript drafting. RC-R: *in vivo* study: critical review analysis, manuscript drafting. AG: *in vivo* study: RTPCR and analysis, manuscript drafting. DG: *in vivo* study: RTPCR, manuscript drafting. JH-J: research project delineation, immunohistochemistry analysis, manuscript final review. EE: *in vivo* study: critical review analysis, manuscript drafiting. JB: research project delineation; manuscript final review. DL: *in vitro* study, research project delineation, manuscript final review. CC: research project delineation, study coordinator, manuscript drafting, manuscript final review.

### Conflict of Interest Statement

The authors declare that the research was conducted in the absence of any commercial or financial relationships that could be construed as a potential conflict of interest.

## Supplementary Material

Figure S1 Immunoperoxidase staining control in histological sections of adult rat cerebral cortex with mechanical brain injury from 48 h postoperative period group. **(A–B')**, Pre-adsorption with Ten-2 primary polyclonal antibody and Ten-2 epitope (**A–A'**, 1:1; **B–B'**, 1:0.01). Observe absence of immunoreaction in neurons and reactive astrocytes in the area with mechanical injury of the cerebral cortex. **(C–C')** Ten-2 primary polyclonal antibody omission, resulting in absence of immunoreaction in neurons and reactive astrocytes. Ab1 (Ten-2), Ten-2 primary antibody omission; ADS, adsorption test; ^*^, cortical lesion area.

Figure S2 Immunoperoxidase staining to identify Ten-1, Ten-3 or Ten-4 in histological sections of adult rat cerebral cortex with mechanical brain injury from 48 h postoperative period group (A-C). Observe only neurons (arrow) exhibiting immunoreactivity to Ten-1 **(A–A”)**. Ten-3-LI **(B–B”)** or Ten-4-LI **(C–C”)** cells are not observed in the cerebral cortex. Ten-1-LI, Ten-1-like immunoreactive; Ten-3-LI, Ten-3-like immunoreactive; Ten-4-LI, Ten-4-like immunoreactive; ^*^, cortical lesion area.

Figure S3 Reactive astrocyte from adult rat cerebral cortex with mechanical brain injury (48 h after lesion) analyzed by confocal microscopy showing nucleus DAPI staining (**A**, blue fluorescence), immunoreactivity to GFAP (**B**, DTAF—green fluorescence), immunoreactivity to Ten-2 (**C**, Cy^3^ immunostaining—red fluorescence) and simultaneous labeling (**D**, merge). Ten-2-LI reactive astrocyte exhibited a punctiform pattern, mainly distributed in the cytosol and occasionaly associated with plasmatic membrane.

Figure S4 Control reactions using conventional PCR, total RNA extracted from cerebral cortex of all experimental groups (*n* = 4, four animals per experimental group) and Ten-2, TCAP-2 and neurofilament light (NFL) primers, visualized in 1.5% agarose gel stained with bromide ethidium. No bands were observed to Ten-2 **(A)**, TCAP-2 **(B)** and NFL **(C)** indicating no significant DNA contamination of the RNA samples.

Video S1 3D reconstruction movie in confocal microscopy of a reactive astrocyte from adult rat cerebral cortex with mechanical brain injury (48 h after lesion) shown in Figure S3.

## Abbreviations

Ab1: primary antibody
Ab1(LPHN-1): LPHN-1 primary antibody omission; Ab1(Ten-2) Ten-2 primary antibody omission
aCSF: artificial cerebrospinal fluid
ADS: adsorption
AM: fluo-4 acetomethyl
ANOVA: analysis of variance
BBB: blood-brain barrier
BDNF: brain-derived neurotrophic factor
Ca^2+^: unbound calcium
CaCl_2_2H_2_O: calcium chloride dihydrate
CCD: camera-coupled device
cDNA: complementary DNA
CEUA: Institutional Committee of Animal Welfare
CNS: central nervous system
CRF: corticotrophin-releasing factor
Cy^3^: cyanine
DAB: diaminobenzidine
DAPI, 4': 6-diamidino-2-phenylindole
DGC: dystrophin-dystroglycan complex
ddH_2_O: double-distilled water
DEPC: diethyl pyrocarbonate
DMEM: Dulbecco's modified eagle's medium
DMSO: dimethyl sulfoxide
DNA: deoxyribonucleic acid
DNase: deoxyribonuclease
dNTP: deoxynucleotides
DOC2: double C2-like domain-containing protein
DTT: DL-Dithiothreitol
EtOH: ethanol
FBS: fetal bovine serum
FGF8: fibroblast growth factor
FITC: fluorescein isothiocyanate
GFAP-LI: glial fibrillary acidic protein-like immunoreactive
HEPES: 4-(2-hydroxyethyl)-1-piperazineethanesulfonic acid
KCl: potassium chloride
Kg: kilograms
LPHN: latrophilin
-LPHN-1, absence of LPHN-1 antibody/ LPHN-1-LI: latrophilin-1-like immunoreactive
LPHN-2-LI: latrophilin-2-like immunoreactive
LPHN-3-LI: latrophilin-3-like immunoreactive
μg: microgram
μL: microliter
MAPK: mitogen-activated protein kinase
mL: milliliter
mg: milligram
MgCl_2_: magnesium chloride
MgCl_2_6H_2_O: magnesium chloride hexahydrate
Mm: millimol
mRNA: messenger RNA
n: number
NaCl: sodium chloride
NFL: neurofilament light
nM: nanomol
PBS: phosphate-buffered saline
PBS-T: phosphate-buffered saline and triton X-100
PCR: polymerase chain reaction
RCF: relative centrifugal force
RNA: ribonucleic acid
RNase: ribonuclease
rpm: revolutions per minute
RT-PCR: reverse transcription polymerase chain reaction
S100B: S100 calcium-binding protein B
SEM: standard error of the mean
TCAP: teneurin C-terminal-associated peptides
TCAP-1: teneurin C-terminal-associated peptide 1
TCAP-2: teneurin C-terminal-associated peptide 2
TCAP-3: teneurin C-terminal-associated peptide 3
TCAP-4, teneurin C-terminal-associated peptide 4;Ten-1: teneurin-1
Ten-2: teneurin-2
Ten-3: teneurin-3
Ten-4: teneurin-4
Ten-2-LI: teneurin-2-like immunoreactive
Uv: ultraviolet.
