# Supplementary figures and images for: A Putative Role of Teneurin-2 and Its Related Proteins in Astrocytes
Source: Front Neurosci. 2019 Jun 27;13:655. doi: 10.3389/fnins.2019.00655 (PMC6609321; doi:10.3389/fnins.2019.00655)

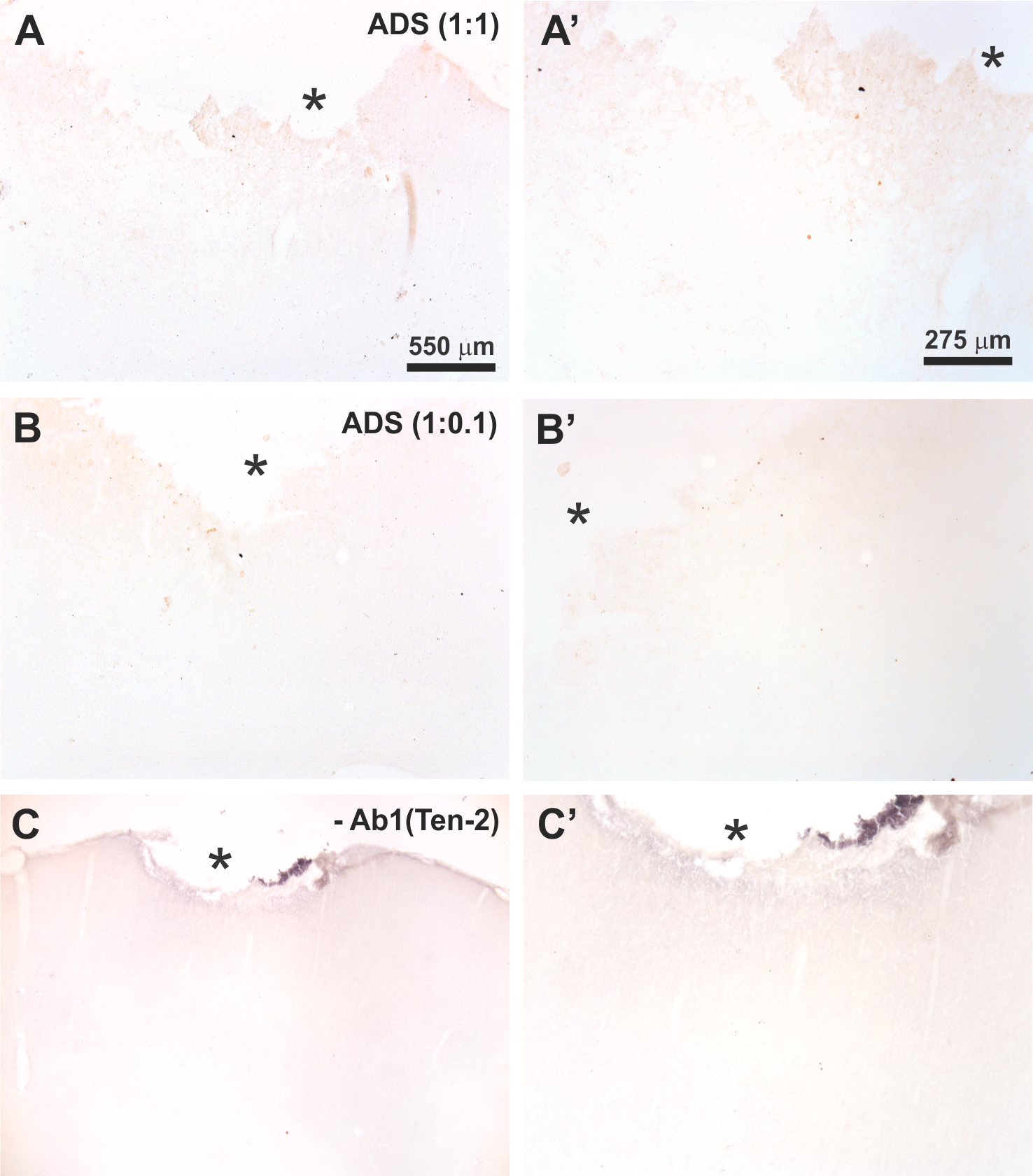

Supplement: Figure S1 — Immunoperoxidase staining control in histological sections of adult rat cerebral cortex with mechanical brain injury from 48 h postoperative period group. (A–B'), Pre-adsorption with Ten-2 primary polyclonal antibody and Ten-2 epitope (A–A', 1:1; B–B', 1:0.01). Observe absence of immunoreaction in neurons and reactive astrocytes in the area with mechanical injury of the cerebral cortex. (C–C') Ten-2 primary polyclonal antibody omission, resulting in absence of immunoreaction in neurons and reactive astrocytes. Ab1 (Ten-2), Ten-2 primary antibody omission; ADS, adsorption test; *, cortical lesion area. [file Image_1.TIF]

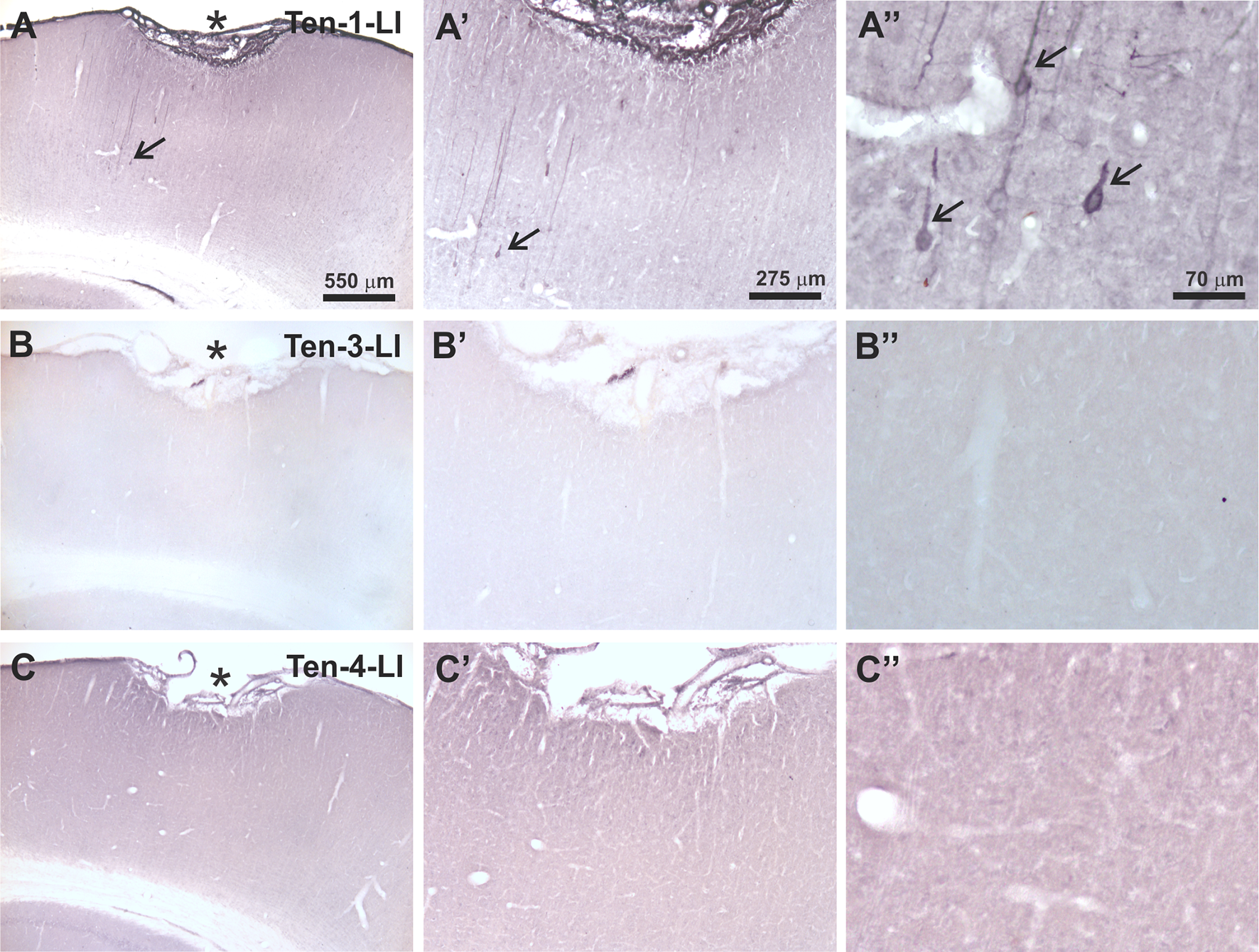

Supplement: Figure S2 — Immunoperoxidase staining to identify Ten-1, Ten-3 or Ten-4 in histological sections of adult rat cerebral cortex with mechanical brain injury from 48 h postoperative period group (A-C). Observe only neurons (arrow) exhibiting immunoreactivity to Ten-1 (A–A”). Ten-3-LI (B–B”) or Ten-4-LI (C–C”) cells are not observed in the cerebral cortex. Ten-1-LI, Ten-1-like immunoreactive; Ten-3-LI, Ten-3-like immunoreactive; Ten-4-LI, Ten-4-like immunoreactive; *, cortical lesion area. [file Image_2.TIF]

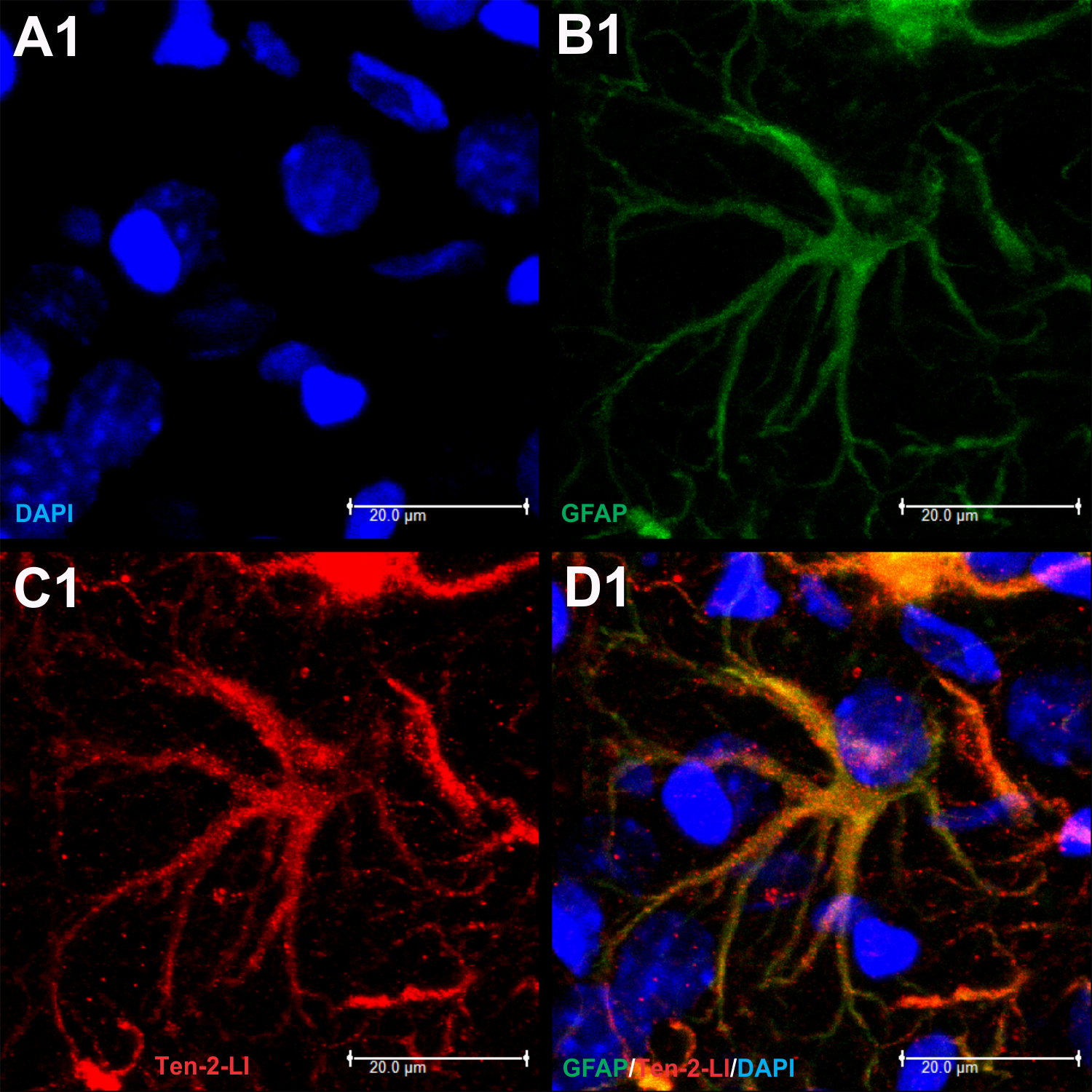

Supplement: Figure S3 — Reactive astrocyte from adult rat cerebral cortex with mechanical brain injury (48 h after lesion) analyzed by confocal microscopy showing nucleus DAPI staining (A, blue fluorescence), immunoreactivity to GFAP (B, DTAF—green fluorescence), immunoreactivity to Ten-2 (C, Cy3 immunostaining—red fluorescence) and simultaneous labeling (D, merge). Ten-2-LI reactive astrocyte exhibited a punctiform pattern, mainly distributed in the cytosol and occasionaly associated with plasmatic membrane. [file Image_3.TIF]

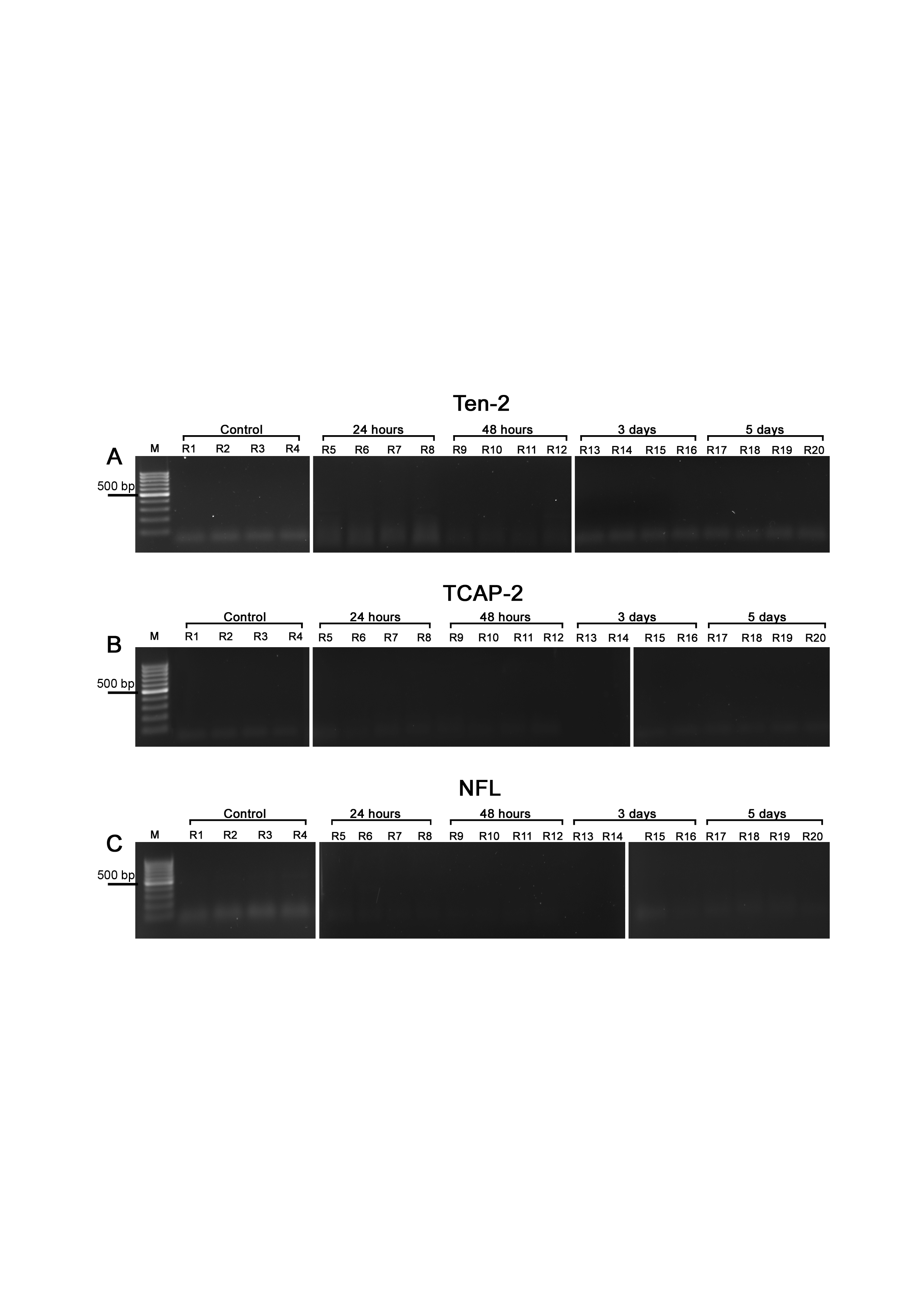

Supplement: Figure S4 — Control reactions using conventional PCR, total RNA extracted from cerebral cortex of all experimental groups (n = 4, four animals per experimental group) and Ten-2, TCAP-2 and neurofilament light (NFL) primers, visualized in 1.5% agarose gel stained with bromide ethidium. No bands were observed to Ten-2 (A), TCAP-2 (B) and NFL (C) indicating no significant DNA contamination of the RNA samples. [file Image_4.TIF]
